# Endovascular thrombectomy for distal medium vessel occlusion stroke: A meta-analysis of randomized controlled trials

**DOI:** 10.1007/s10143-025-03835-0

**Published:** 2025-10-10

**Authors:** Ali Mortezaei, Nadir Al-Saidi, Khaled M. Taghlabi, Ibrahim Mohammadzadeh, Bardia Hajikarimloo, Mohammad Amin Habibi, Robert W. Regenhardt, Joshua S. Catapano, Jan-Karl Burkhardt, Redi Rahmani, Adam A. Dmytriw, Amir H. Faraji, Vivek S. Yedavalli, Visish M. Srinivasan

**Affiliations:** 1 Student Research Committee, Gonabad University of Medical Sciences, Gonabad, Iran; 2 College of Medicine, Central Michigan University, Mount Pleasant, MI USA; 3 Clinical Innovations Laboratory, Houston Methodist Research Institute, Houston, TX USA; 4 Department of Neurological Surgery, Houston Methodist Hospital, Houston, TX USA; 5 Skull Base Research Center, Loghman-Hakim Hospital, Shahid Beheshti University of Medical Sciences, Tehran, Iran; 6 Department of Neurological Surgery, University of Virginia, Charlottesville, VA USA; 7 Department of Neurosurgery, Shariati Hospital, Tehran University of Medical Sciences, Tehran, Iran; 8 Department of Neurology, Massachusetts General Hospital, Harvard Medical School, Boston, MA USA; 9 Department of Neurosurgery, Perelman School of Medicine, University of Pennsylvania, 3400 Civic Center Blvd, Philadelphia, PA 19104 USA; 10 Neuroendovascular Program, Massachusetts General Hospital & Brigham and Women’s Hospital, Harvard Medical, School, Boston, MA 02114 USA; 11 Department of Radiology and Radiological Sciences, The Johns Hopkins Hospital, Baltimore, MD USA

**Keywords:** Distal medium vessel occlusion, DMVO, Endovascular thrombectomy, Acute ischemic stroke

## Abstract

The efficacy of endovascular thrombectomy (EVT) for acute ischemic stroke (AIS) resulting from distal medium vessel occlusion (DMVO) remains uncertain. This systematic review and meta-analysis aimed to provide a comprehensive assessment of EVT in AIS due to DMVO, based on evidence derived from randomized controlled trials (RCTs). We systematically reviewed four electronic databases to find relevant studies. Risk ratios (RRs) and mean differences (MDs) with 95% confidence intervals (CIs) were calculated for categorical and continuous outcomes, respectively. Significant heterogenicity was resolved with leave-one-out influence analysis. There were 601 patients in the EVT plus best medical treatment (BMT) group and 623 patients in the BMT alone group. Findings showed a significantly higher rate of serious adverse events (RR = 1.3, 95%CI: 1.1–1.5, *P* < 0.01) and symptomatic intracranial hemorrhage (RR = 3.3, 95%CI: 1.8–5.9, *P* < 0.01) in thrombectomy than the control group. There was no significant difference between two groups in 90-day functional excellence (mRS 0–1), functional independence (mRS 0–2), and mortality. Subgroup analysis, including the three DMVO trials and the two secondary analyses of previous LVO RCTs, confirmed the prior findings. Leave-one-out influence analysis showed no significant heterogeneity in any primary and secondary outcomes. Our findings indicate that while EVT does not provide a significant advantage over BMT alone in improving functional outcomes for patients with DMVO, it is associated with an increased risk of hemorrhagic complications. These results underscore the importance of a cautious approach when considering EVT for DMVO and emphasize the need for further RCTs to optimize treatment strategies.

**Supplementary Information:**

The online version contains supplementary material available at 10.1007/s10143-025-03835-0.

## Introduction

The treatment for acute ischemic stroke (AIS) has been revolutionized over the past decade by the introduction of mechanical endovascular thrombectomy (EVT), which has been proven to be particularly beneficial for large-vessel occlusions (LVOs) [1–4]. Until recently, EVT was also considered by many to have a role in the management of strokes involving medium and distal vessels too, backed by evidence from observational studies and subgroup analyses of previous trials focused on LVOs [5, 6]. However, recent randomized controlled trials (RCTs) were published, DISTAL [7] and ESCAPE-MeVO [8] and presented, DISCOUNT, that became the first to directly compare EVT to best medical treatment (BMT) alone for distal- and medium-occlusions (DMVO), and all three studies reached the same verdict: EVT added no clinical benefit over BMT.

While definitions vary in the literature, DMVO occur in nondominant or codominant segments of M2, M3, and M4 segments of the middle cerebral artery, as well as the anterior cerebral artery, posterior cerebral artery, posterior inferior cerebellar artery, anterior inferior cerebellar artery, and superior cerebellar artery, in contrast to large-vessel occlusions, which involve the M1 segment of the middle cerebral artery, intracranial internal carotid artery, and basilar artery [9]. DMVOs are estimated to account for 25–40% of AISs and are uniquely challenging to treat with EVT because of their smaller vessel size, distal location, increased tortuosity, and variable collateral circulation [9–11]. These factors heighten the procedure’s complexity, potentially increasing the risk for complications and serious adverse events (SAEs) [10]. And, with the relatively lower ischemic burden in these cases, in which a smaller area of the brain is affected compared to LVOs, there may be less potential benefit from reperfusion [12]. For these reasons, in tandem with challenges in overall trial logistics such as the difficulty in enrolling patients in high-powered trials, there was a growing reliance with the results of observational studies post hoc analyses rather than RCTs in the past [13].

However, with a number of RCTs recently published, high-level data challenges previous conclusions. ESCAPE-MeVO [8] trial found that EVT provided no additional benefit over BMT in DMVO, with no significant difference in functional independence between groups, while also suggesting higher rates of symptomatic intracranial hemorrhage (sICH) (5.4% vs. 2.2%) and mortality (13.3% vs. 8.4%). Similarly, DISTAL trial [7] concluded that EVT did not reduce disability or death compared to BMT alone for treatment of DMVO, with consistent findings across subgroups, including patients with moderate to severe strokes and those who did not receive intravenous thrombolysis. Further consistent with these findings, the DISCOUNT trial [14] conducted across 22 French university hospitals, was terminated early after an interim analysis of 163 patients revealed no superiority of EVT over BMT alone for DMVO alongside a higher rate of intracranial hemorrhage (12% vs. 6%).

Thus, a pooled analysis is essential in resolving ongoing clinical uncertainty and informing treatment guidelines to ensure that stroke patients receive interventions based on the strongest available evidence. This systematic review and meta-analysis aimed to clarify whether EVT confers any measurable benefit over BMT in the treatment of DMVO and to reconcile these results with previous observational studies that suggested a positive effect.

## Methods

### Search strategy and selection process

Inclusion criteria were (1) RCTs, (2) trials that compared EVT plus BMT versus BMT alone for DMVO stroke (DMVO was defined as an occlusion involving the nondominant or codominant M2 segment of the middle cerebral artery [MCA]; occlusion of M3 or M4 segments of the MCA; occlusion of A1, A2, or A3 segments of the anterior cerebral artery [ACA]; or occlusion of P1, P2, or P3 segments of the posterior cerebral artery [PCA]), (3) AIS was defined as an acute ischemic stroke resulting from an isolated DMVO, as confirmed through computed tomographic (CT) angiography or magnetic resonance (MR) angiography, (4) admission National Institutes of Health Stroke Scale (NIHSS) ≥ 4, (5) participants aged ≥ 18 years, and 4) patients presented within 24 h of last known well to randomization. Exclusion criteria consisted of (1) case reports, observational studies, reviews, and editorials, (2) LVO stroke, and (3) single-arm studies.

### Search strategy and selection process

We performed a comprehensive search across PubMed/MEDLINE, Scopus, Web of Science, Cochrane Central Register of Controlled Trials (CENTRAL), and ClinicalTrials.gov. The search covered all records up to February 11, 2024 adhered to Preferred Reporting Items for Systematic Reviews and Meta-Analyses (PRISMA) guidelines. Search strategy incorporated terms such as “endovascular thrombectomy”, “distal or medium vessel occlusion”, “best medical treatment”, “acute ischemic stroke”, and “randomized controlled trials” to identify relevant studies. To ensure thoroughness of bibliography, we also conducted a manual review of reference lists from relevant published studies and international conference abstracts.

### Screening process

Two authors (A.M. and N.A.S.) independently conducted the screening process based on predefined eligibility criteria. During both the title/abstract and full-text screening phases, a third author (R.R.) was involved in resolving conflicts and ensuring selection process consistency.

### Data extraction

Two authors (N.A.S. and K.M.T.) independently collected data using a standardized Excel spreadsheet, adhering strictly to the full text of the RCTs and their supplementary online appendix. To ensure accuracy and completeness, a third author (A.M.) conducted a thorough review of the extracted data. Any disparities were addressed through team discussions, with unresolved discrepancies adjudicated by a third-party reviewer (R.R.) when necessary.

### Outcomes measure

The primary outcome was functional independence (modified [mrs]ankin scale [mRS] 0–2) at 90 days Secondary outcomes included functional excellence (mRS 0–1), independent ambulation (mRS 0–3), death or dependency (mRS score 4–6), mortality at three months, successful recanalization (modified treatment in cerebral infarction [mTICI] score of 2b-3), serious adverse events (SAE), and sICH (was defined as an increase of ≥ 4 points on the NIHSS, due to cerebral hemorrhage detected on imaging within 36 h of symptom onset.) [15, 16].

### Quality and risk of bias assessment

The quality of the included studies was assessed using the Cochrane Collaboration’s Risk of Bias 2 (ROB2) tool for randomized trials. Two independent authors (A.M. and K.M.T.) evaluated various domains, including random sequence generation, allocation concealment, blinding of participants and personnel, blinding of outcome assessment, completeness of outcome data, selective reporting, and other potential sources of bias and any disparities were resolved through consensus with the team.

### Statistical analysis

A meta-analysis was performed using the “netmeta” package in R version 4.3.0. For categorical outcomes, risk ratios (RRs) with 95% confidence intervals (CIs) were calculated, while mean differences (MDs) were used for continuous outcomes. Statistical heterogeneity was considered significant based on α < 0.1 and I² >50%, following Cochrane handbook recommendations [17]. Depending on level of heterogeneity, either a fixed-effect or random-effects model was applied. Leave-one-out sensitivity analyses were conducted to evaluate robustness of findings by excluding trials contributing to high heterogeneity or exhibiting a high risk of bias. Analysis was repeated after omitting these studies to ensure homogeneity. Subgroup analyses were conducted based on key clinical and methodological characteristics to identify and resolve potential sources of heterogeneity. Median and range values were converted to mean and standard deviation (SD) using method proposed by Luo et al. [18].

## Results

**Fig. 1:**
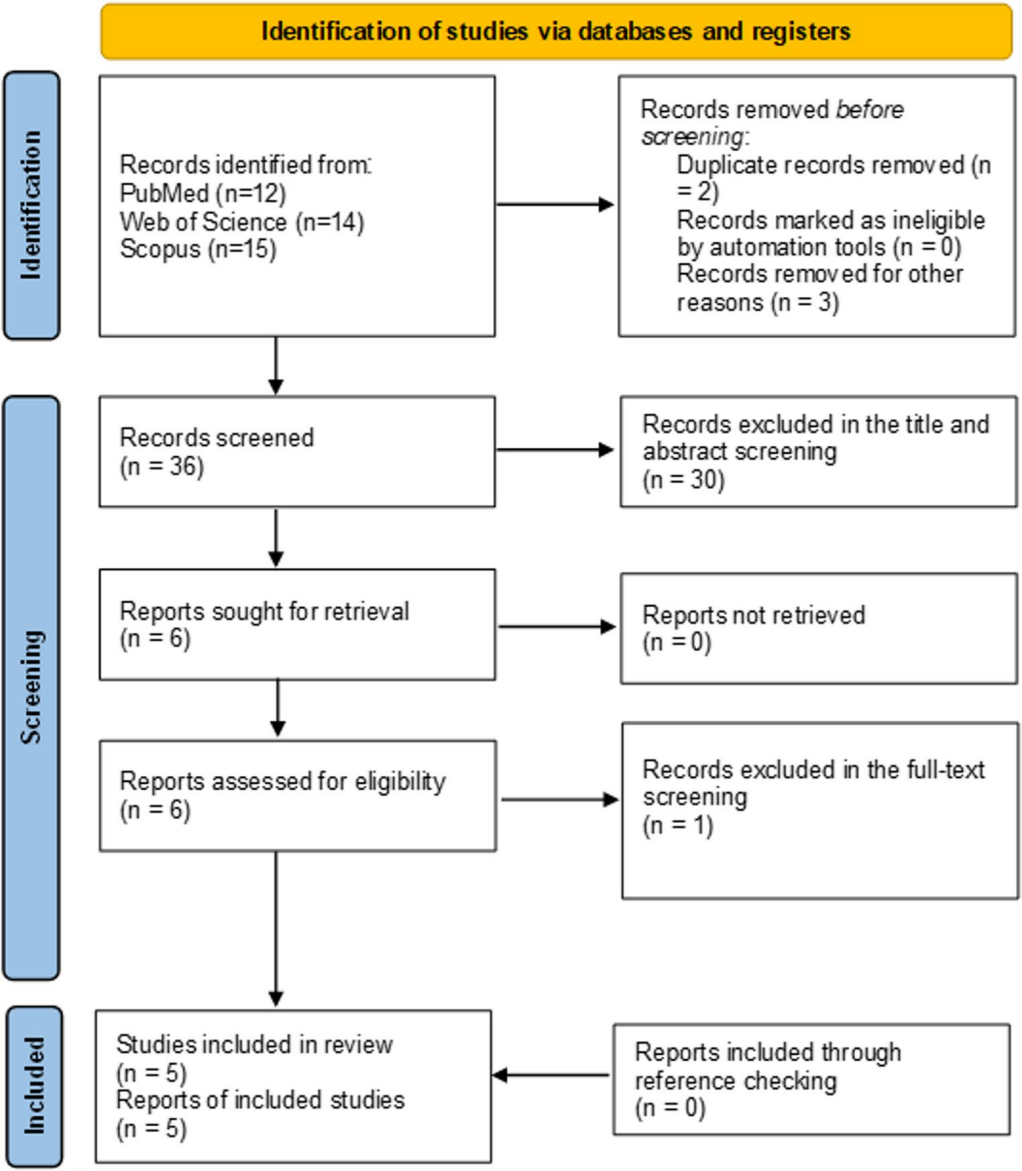
PRISMA study selection flow chart

Study selection 41 records were found in systematic initial literature search. After removal of duplicate studies, title/abstract and full-text screening, three RCTs [7, 8, 14] assessed EVT plus BMT versus BMT Alone were included in analysis. Additionally, we included two secondary analyses of previous LVO RCTs only for DMVO subgroup analysis [6, 19]. For outcomes reported in all three identified RCTs [7, 8, 14] (DISTAL, ESCAPE-MeVO, and DISCOUNT), we re-analyzed outcomes of interest excluding DISCOUNT [14] and only focus on two major published RCTs [7, 8]. There was substantial reliability for study selection for both the title/abstract (Cohen’s κ = 0.96) and the full-text screening steps (Cohen’s κ = 0.99) [20]. PRISMA study selection flowchart is displayed in Fig. 1.

Study characteristics and baseline study demographic A detailed assessment of characteristics of each RCT is provided in Table 1. There were 601 patients in EVT plus BMT group and 623 patients in BMT alone group. ESCAPE-MeVO [8] trial did not included patients with A1 and P1 occlusion while DISTAL [7] and DISCOUNT [14] included these patients. DISTAL [7] and DISCOUNT [14] included patients based on non-perfusion imaging modalities (Table 1). Baseline demographic and characteristics were reported in Table 2. There is well balanced baseline characteristics across included variables.

**Table 1:** Characteristics of the all included trials

Study	Recruiting centers	Time window	Eligibility criteria			
			Imaging	NIHSS	Age of patients	Location of occlusion
DISTAL	International	Up to 6 h of LSW or between 6 and 24 h of imaging indicated the presence of salvageable tissue.	Isolated DMVO on CTA/MRA	≥ 4	≥ 18	MCA: nondominant or codominant M2, M3, and M4 segments ACA: A1, A2, and A3 segments PCA: P1, P2, and P3 segments
ESCAPE-MeVO	International	Up to 12 h of LSW and had favorable baseline imaging.	Isolated DMVO on CT/MRI or CTA/MRA or CT/MRI perfusion	≥ 5	≥ 18	MCA: M2 and M3 segments ACA: A2, and A3 segments PCA: P2, and P3 segments
DISCOUNT	France	Interval between symptom onset and expected groin puncture ≤ 8 h or LSW up to 24 h without any hyperintense signal on FLAIR	Isolated DMVO on CTA or MRI	≥ 5	≥ 18	MCA: Distal M2 (above the mid-insular level), and M3 segments ACA: A1, A2, and A3 segments PCA: P1, P2, and P3 segments
Qureshi et al., 2017	Turkey	Mean (SD) of LSW to randomization: 2.45 (0.62) in intervention group and 2.36 (1.28) in control group†	Isolated M2 occlusion on CTA/MRA	14 (8–24) ‡	18–82	M2
Menon et al., 2019	International	Mean (SD) of LSW to randomization: 3.33 (1.4) in intervention group and 3.48 (1.28) in control group†	Isolated M2 occlusion on CTA/MRA	15 (11–20) ‡	≥ 18	M2

DISTAL, Endovascular Therapy plus Best Medical Treatment (BMT) versus BMT Alone for Medium Vessel Occlusion Stroke — A Pragmatic, International, Multicenter, Randomized Trial; ESCAPE-MeVO, Endovascular Treatment to Improve Outcomes for Medium Vessel Occlusions; DISCOUNT, Evaluation of Mechanical Thrombectomy in Acute Ischemic Stroke Related to a Distal Arterial Occlusion; EVT, endovascular thrombectomy; BMT, best medical treatment; NIHSS, National Institutes of Health Stroke Scale; MCA, middle cerebral artery; ACA, anterior cerebral artery; PCA, posterior cerebral artery; LSW, last seen well; DMVO, distal medium vessel occlusion stroke; FLAIR, Fluid-attenuated inversion recovery; CTA, computed tomography angiography; MRA, magnetic resonance angiography;

† Specific time window was not reported in inclusion criteria

‡ Specific NIHSS cutoff was not provided, data reported as median (IQR)

**Table 2:** Detailed baseline demographic and characteristics of the included patients

Variable	EVT plus BMT	BMT Alone
Patient age, mean (SD), y	76.19 (11.55)	73.8 (13.78)
Females	234/526 (44.5%)	250/546 (45.8%)
Past medical history		
Hypertension	363/526 (69%)	388/544 (71.3%)
Hyperlipidemia	229/526 (43.5%)	252/544 (46.3%)
Diabetes mellitus	124/526 (23.6%)	140/544 (25.7%)
Atrial fibrillation	149/526 (28.3%)	145/544 (26.7%)
Previous stroke	122/526 (23.2%)	115/544 (21.1%)
Vessel occlusion location†		
M2	256/524 (48.8%)	209/541 (38.6%)
M3	152/524 (29%)	210/541 (38.8%)
A2	15/524 (2.9%)	14/541 (2.6%)
A3	17/524 (3.2%)	8/541 (1.5%)
P2	55/524 (10.5%)	67/541 (12.4%)
P3	6/524 (1.1%)	17/541 (3.1%)
IV-tPA usage	312/526 (59.3%)	352/546 (64.7%)
NIHSS at admission, mean (SD)	7.54 (3.41)	7.20 (3.78)
Last seen well to randomization, mean (SD)	4.97 (4.29)	4.94 (4.51)

EVT, endovascular thrombectomy; BMT, best medical treatment; NIHSS, National Institutes of Health Stroke Scale; IV-tPA, intravenous tissue plasminogen activator;

†M2 and M3 segments of middle cerebral artery; A2 and A3 segments of anterior cerebral artery; P2 and P3 segments of posterior cerebral artery;

### Functional and efficacy outcomes

#### Excellent functional outcome at 90 days

Two RCTs [7, 8] with a total of 1069 patients were included for analysis of excellent functional outcome (mRS 0–1) at 90 days. Findings showed that there was no significant difference (RR = 0.95, 95%CI: 0.82–1.1, *P* = 0.46) in 90-day mRS 0–1 between thrombectomy plus medical treatment and medical treatment alone (eFigure1 in Supplement). Notably, there was no heterogeneity among the studies (I^2^ = 0.0, *P* = 0.77) (Table 3).

**Table 3:** Comparisons of functional and efficacy outcomes between two groups

Variable	EVT plus BMT	BMT Alone	Effect size, RR	95% CI	*P*	I^2^	τ^2^	H-statistics	Leave-One-Out
Excellent functional outcome at 90 days†	200/526 (38%)	219/543 (40.3%)	0.95	[0.82 - 1.1]	0.46	0.00	0.00	1.00	NS
Independent functional outcome at 90 days‡	336/601 (55.9%)	367/620 (59.2%)	0.95	[0.86–1.04]	0.25	52.0	0.001	1.44	NS
Ambulatory functional outcome at 90 days	384/526 (73%)	417/543 (76.8%)	0.95	[0.9–1.02]	0.15	0.00	0.00	1.00	NS
Death or dependency at 90 days	142/526 (27%)	126/543 (23.3%)	1.16	[0.95–1.43]	0.15	0.00	0.00	1.00	NS
Overall survival at 90 days‡	512/590 (86.8%)	572/640 (89.4%)	0.97	[0.94–1.02]	0.23	53.8	0.001	1.47	NS
Mortality at 90 days‡	78/590 (13.2%)	68/640 (10.6%)	1.21	[0.9 - 1.6]	0.23	34.8	0.09	1.24	NS
Successful recanalization§	370/504 (73.4%)	NA	73.4%¶	[70% – 77%]	NA	0.00	0.00	1.00	NS

EVT, endovascular thrombectomy; BMT, best medical treatment; RR, risk ratio; CI, confidence interval; NS, not significant; Sig, significant;

† Excellent functional outcome was defined as mRS 0-1, independent functional outcome was defined as mRS 0-2, ambulatory functional outcome was defined as mRS 0-3, death or dependency was defined as mRS 4-6, and overall survival was defined as mRS 0-5

‡ Re-analysis excluding DISCOUNT trial for 90-day functional independence (RR=0.98, 95%CI: 0.9 - 1.1, *P*=0.65, I^2^=10.5%), overall survival (RR=0.96, 95%CI: 0.9 - 1.0, *P*=0.12, I^2^=0.0%), and mortality rate (RR=1.28, 95%CI: 0.9 - 1.7, *P*=0.13, I^2^=21.1%) confirmed the previous findings

§Successful recanalization was defined as modified treatment in cerebral infarction (mTICI) score of 2b-3

¶Analysis was performed using proportion meta-analysis

#### Independent functional outcome at 90 days

Rate of functional independence (mRS 0–2) were reported for 1221 patients across three RCTs [7, 8, 14]. The rate of 90-day mRS 0–2 was not significantly differ (RR = 0.95, 95%CI: 0.82–1.1, *P* = 0.46) between EVT plus BMT versus BMT alone with non-significant heterogeneity among studies (I^2^ = 52.0, *P* = 0.13) (Table 3) (eFigure2 in Supplement). Additional analysis on only DISTAL [7] and ESCAPE-MeVO [8] trials confirmed previous findings (RR = 0.98, 95%CI: 0.9–1.1, *P* = 0.65, I^2^ = 10.5%).

#### Ambulatory functional outcome at 90 days

Meta-analysis of 90-day ambulatory functional outcome (mRS 0–3) across two RCTs [7, 8] showed no significant difference (RR = 0.95, 95%CI: 0.9–1.02, *P* = 0.15) between two groups. Importantly, there no heterogeneity across trials (I^2^ = 0.0, *P* = 0.50) (Table 3) (eFigure3 in Supplement).

#### Death or dependency at 90 days

Two trials [7, 8] were included for analysis of death or dependency (mRS 4–6) at three months. Results showed no significant difference (RR = 1.16, 95%CI: 0.95–1.43, *P* = 0.15) in 90-day mRS 4–6 between two groups with no heterogeneity (I^2^ = 0.0, *P* = 0.50) (Table 3) (eFigure4 in Supplement).

#### Overall survival at 90 days

Rate of 90-day overall survival (mRS 0–5) was reported for 1230 patients in three trials [7, 8, 14]. Overall survival at three months was 86.6% (512 out of 590) for thrombectomy group and 89.4% (572 out of 640) for BMT alone group. Analysis showed no significant difference in 90-day overall survival (RR = 0.97, 95%CI: 0.94–1.02, *P* = 0.23) between two group with moderate non-significant heterogeneity (I^2^ = 53.8, *P* = 0.12). Additional analysis on only DISTAL [7] and ESCAPE-MeVO [8] trials showed similar results **(**RR = 0.96, 95%CI: 0.9–1.0, *P* = 0.12, I^2^ = 0.0%**)** (Table 3) (eFigure5 in Supplement).

#### 90-day mortality

Results from 3 trials [7, 8, 14] involving 1230 patients revealed that occurrence of mortality was numerically higher in EVT plus BMT group (78 of 590 [13.2%]) compared with BMT alone group (68 of 640 [10.6%]). However, the calculated RR of 1.21 (95% CI, 0.9–1.6, *P* = 0.13) did not reach statistical significance (eFigure6 in Supplement). Notably, there was minimal non-significant heterogeneity among the studies included (I^2^ = 34.8, *P* = 0.21). Further analysis excluding DISCOUNT [14] trial reduced heterogeneity and showed similar findings (RR = 1.28, 95%CI: 0.9–1.7, *P* = 0.13, I^2^ = 21.1%) (Table 3).

### Safety outcomes

#### Serious adverse events

We included three RCTs [7, 8, 14] involving 1230 patients for assessing 90-day SAE. Occurrence of 90-day SAE was higher in thrombectomy group (226 of 592 [38.2%]) compared with control group (188 of 638 [29.5%]). The difference between two group was statistically significant (RR = 1.3, 95%CI: 1.1–1.5, *P* **< **0.01) and favored BMT alone group, without any heterogeneity (I^2^ = 0.0, *P* = 0.98) (eFigure7 in Supplement). A re-analysis excluding DISCOUNT [14] trial confirmed prior findings (RR = 1.3, 95%CI: 1.1–1.53, *P* = 0.003, I^2^ = 0.0%) (Table 4).

**Table 4:** Comprehensive assessment of safety outcomes between two groups

Variable	EVT plus BMT	BMT Alone	Effect size, RR	95% CI	*P*	I^2^	τ^2^	H-statistics	Leave-One-Out
Serious adverse events at 90 days†	226/592 (38.2%)	188/638 (29.5%)	1.3	[1.1–1.5]	**< 0.01**	0.00	0.00	1.00	**Sig**
Any serious adverse events									
Pneumonia	31/528 (5.9%)	14/544 (2.6%)	2.3	[1.2–4.3]	**0.009**	0.00	0.00	1.00	NS
Ischemic Stroke	5/528 (0.95%)	1/544 (0.2%)	1.07	[0.6–1.80]	0.8	17.0	0.03	1.1	NS
Infection	17/528 (3.2%)	8/544 (1.5%)	2.2	[0.95–5.03]	0.065	0.00	0.00	1.00	NS
Seizure	4/528 (0.76%)	7/544 (1.3%)	0.6	[0.18–2.02]	0.41	0.00	0.00	1.00	NS
Endocarditis	5/528 (0.95%)	1/544 (0.2%)	5.2	[0.6–45.2]	0.13	0.00	0.00	1.00	NS
Cardiac Failure	5/528 (0.95%)	8/544 (1.5%)	0.65	[0.2–1.96]	0.44	0.00	0.00	1.00	NS
Heidelberg intracranial hemorrhage classification									
Hemorrhagic infarction type 1	70/528 (13.3%)	74/540 (13.7%)	1.1	[0.8–1.5]	0.58	0.00	0.00	1.00	NS
Hemorrhagic infarction type 2	39/528 (7.4%)	22/540 (4.1%)	1.8	[1.1–3.03]	**0.019**	0.00	0.00	1.00	NS
Parenchymal hematoma type 1	29/528 (5.5%)	19/540 (3.5%)	1.6	[0.9–2.8]	0.12	0.00	0.00	1.00	NS
Parenchymal hematoma type 2	18/528 (3.4%)	13/540 (2.4%)	1.4	[0.7–2.8]	0.35	0.00	0.00	1.00	NS
Intraventricular hemorrhage	6/528 (1.1%)	5/540 (0.93%)	1.21	[0.4–3.9]	0.75	0.00	0.00	1.00	NS
Subarachnoid hemorrhage†	43/691 (6.2%)	15/635 (2.8%)	3.3	[1.8–5.9]	**< 0.01**	44.3	0.35	1.34	NS

EVT, endovascular thrombectomy; BMT, best medical treatment; RR, risk ratio; CI, confidence interval; NS, not significant; Sig, significant;

†Re-analysis excluding DISCOUNT trial for 90-day serious adverse events at 90 days (RR=1.3, 95%CI: 1.1 - 1.53, *P*=0.003, I^2^=0.0%) showed similar findings, and for subarachnoid hemorrhage (RR=4.2, 95%CI: 0.0 – 46027.4, *P*=0.3, I^2^=66.2%) the difference got non-significant

Meta-analysis of two RCTs [7, 8] on specific SAEs showed a significantly higher rate of pneumonia (RR = 2.3, 95%CI: 1.2–4.3, *P* = 0.009, I2 = 0.0%) in EVT plus BMT than BMT Alone, with no statistical differences in occurrence of ischemic stroke, infection, seizure, endocarditis, and cardiac failure (Table 4).

#### Intracranial hemorrhages

Three trials [7, 8, 14] that evaluated sICH were included in analysis. Findings showed that rate of sICH was significantly higher (RR = 3.3, 95%CI: 1.8–5.9, *P* < 0.01) in thrombectomy group (43 of 691 [6.2%]) than BMT alone (15 of 635 [2.8%]), with non-significant heterogeneity (I^2^ = 44.3, *P* = 0.17) (eFigure8 in Supplement). Importantly, re-analysis on only DISTAL [7] and ESCAPE-MeVO [8] trials showed no significant difference in sICH (RR = 4.2, 95%CI: 0.0–46027.4, *P* = 0.3, I^2^ = 66.2%) between two groups (Table 4).

Meta-analysis of two RCTs [7, 8] on other intracranial hemorrhages showed a significantly higher rate of hemorrhagic infarction type 2 (RR = 1.8, 95%CI: 1.1–3.03, *P* = 0.019, I^2^ = 0.0%) in intervention group compared to BMT group, without any significant differences in other intracranial hemorrhages (Table 4).

### Subgroup analysis

Meta-analysis including three DMVO RCTs [7, 8, 14] and two secondary analyses of previous LVO RCTs [6, 19] confirmed previous findings and showed no significant differences between two groups in 90-day mRS 0–1 (RR = 1.15, 95%CI: 0.6–2.1, *P* = 0.49) (I^2^ = 54.1, *P* = 0.09), 90-day mRS 0–2 (RR = 1.01, 95%CI: 0.8–1.35, *P* = 0.93) (I^2^ = 61.3, *P* = 0.04), 90-day mRS 0–3 (RR = 0.99, 95%CI: 0.9–1.05, *P* = 0.66) (I^2^ = 49.8, *P* = 0.11), sICH (RR = 1.5, 95%CI: 0.2–11.2, *P* = 0.6) (I^2^ = 65.3, *P* = 0.02), and mortality (RR = 0.85, 95%CI: 0.3–2.4, *P* = 0.69) (I^2^ = 58.6, *P* = 0.045).

### Leave-one-out sensitivity analysis

There was no significant heterogeneity among all primary and secondary outcomes. However, significant heterogeneity was observed in the subgroup analyses. Sensitivity analyses successfully resolved heterogeneity in all analyses except for sICH. Notably, despite resolving heterogeneity, all subgroup results remained non-significant.

### Quality assessment

Our risk of bias assessment for included trials using ROB2 revealed a low risk of bias among two studies and moderate risk in three studies (eFigures 9 and 10 in Supplement). Notably, domains showed minor deviations from intended interventions and selection process.

## Discussion

Our meta-analysis of RCTs evaluating EVT versus BMT for patients with confirms what each individual trial concluded, that there was no significant difference between EVT plus BMT vs. BMT alone among functional outcomes. Additionally, EVT was associated with a higher rate of various SAE and sICH. Findings showed no significant difference between EVT versus BMT alone in functional independence (55.9% vs. 59.2%; *P* = 0.25), excellent function, ambulatory functional, death or dependency, and mortality (13.2% vs. 10.6%; *P* = 0.23) at 90 days. Furthermore, EVT was associated with a higher rate of SAEs (38.2% vs. 29.5%; RR = 1.3, *P* < 0.01), particularly for pneumonia (5.9% vs. 2.6%; RR = 2.3, *P* = 0.009), sICH (6.2% vs. 2.8%; RR = 3.3, *P* < 0.01), and type 2 hemorrhagic infarctions (7.4% vs. 4.1%; RR = 1.8, *P* = 0.019). Furthermore, subgroup analysis of five RCTs [6–8, 14, 19] confirmed these results. These findings align with conclusions of individual RCTs, reinforcing notion that EVT does not provide a meaningful clinical advantage over BMT for patients with DMVO stroke and may introduce additional risks.

Compared to previous subgroup analyses and observations studies [5, 6, 19, 21, 22] our meta-analysis opposes the notion that EVT affords a benefit in DMVO strokes. One key factor that may explain this discrepancy is inherent selection bias in observational studies. Specifically, many observation studies included patients selectively treated with EVT based on physician judgment [23]. Although RCTs included both anterior and posterior circulation DMVOs, large multicenter studies focusing exclusively on anterior circulation occlusions [24] have corroborated these findings, demonstrating no significant benefit of thrombectomy over conservative management, even when combined with adjuvant intravenous thrombolysis [25]. These findings remained consistent even in studies that excluded patients with M2 segment occlusions [26]. Furthermore, while prior studies reported conflicting findings regarding hemorrhagic risk, our analysis revealed a threefold increase in the risk of sICH among patients undergoing EVT compared to those receiving BMT alone (RR = 3.3, 95%CI: 1.8–5.9, *P* < 0.01). However, a sensitivity analysis excluding the DISCOUNT trial rendered association with sICH nonsignificant, with substantial heterogeneity and a wide confidence interval (RR = 4.2, 95%CI: 0.0–46027.4, *P* = 0.3, I²=66.2%), thereby limiting the robustness of this analysis and suggesting that the observed signal may be trial-specific and should be interpreted cautiously.

According to RCTs, this may have potentially been influenced by the growing knowledge that EVT is beneficial for LVOs and assumption by many that the procedure would show similar impact for DMVO patient population [7, 8, 23]. Additionally, the lack of randomization in previous trials and heterogeneity of occlusion locations and stroke severity in patient populations play equal roles in limitations of previous conclusions. Another consideration is that patients enrolled in the RCTs did not have severely high NIHSS (medians 6–8). Therefore, they do not support routinely withholding EVT from patients with very severe strokes, particularly associated with M2 occlusions. Moreover, the inclusion of anatomically diverse occlusion sites, such as M2, M3, M4, ACA, and PCA, in pooled analyses likely contributes to clinical heterogeneity, as prior evidence suggests that EVT may be beneficial for M2 occlusions, while more distal branches are less likely to show a treatment effect. This particular patient population warrants further study. This may be another potential explanation for contradictory results compared to some observational studies. Moreover, although the difference in mRS 0–2 rates was not statistically significant, the numerically higher proportion of favorable outcomes in the BMT group (3.3%) raises the possibility of differential treatment effects in specific patient subgroups. Furthermore, the high rate of successful recanalization (73.4%) in the EVT group without a corresponding clinical benefit highlights a potential disconnect between technical success and functional outcomes. This paradox warrants further exploration into contributing pathophysiological factors such as microvascular damage, collateral insufficiency, or reperfusion injury.

In addition to the heterogeneity of trial inclusion criteria and patient characteristics, technical factors such as device selection and operator experience may also affect outcomes following EVT for DMVO. A recent meta-analysis [27] demonstrated that the use of stent retrievers, either alone or in combination with aspiration, was associated with improved functional independence and lower mortality compared to aspiration alone, without increasing the risk of sICH. Furthermore, evidence suggests that greater operator experience correlates with shorter procedure times and higher recanalization success, underscoring the importance of technical expertise in optimizing EVT results [28]. These factors likely contribute to the observed variability in clinical outcomes across studies and should be considered in future trials evaluating EVT for DMVO.

### Strength and limitations

Our study analyzed data exclusively from RCTs evaluating EVT plus BMT vs. BMT alone in DMVO. With no significant heterogeneity across any key functional or safety outcomes and a focus on high-quality randomized evidence, our analysis minimizes bias and provides the most reliable assessment of EVT as a treatment modality for this population. By employing standardized primary and secondary outcomes—including 90-day mRS, mortality, and detailed SAE—this study enables meaningful comparisons across included trials, thereby enhancing validity and generalizability of our findings.

Despite these strengths, limitations must be addressed. Most notably, this meta-analysis is restricted to only three RCTs, and while these trials provide us with the highest level of data available and have adequate sample sizes, larger populations could strengthen our conclusions and increase statistical power. Additionally, there was variability between studies regarding inclusion criteria and patient selection. For example, DISTAL allowed EVT up to 24 h from symptom onset, while ESCAPE-MeVO used a stricter 12-hour window, with both trials selecting patients based on favorable MRI imaging criteria. DISCOUNT, on the other hand, imposed an even more restrictive window, permitting EVT within 6 h of symptom onset or up to 24 h only if no hyperintense signal was detected on fluid-attenuated inversion recovery (FLAIR) imaging. Other possible discrepancies between studies include the proportions of different arteries and segments, stroke severity, type of EVT device utilized, anesthesia approach, and, perhaps, operator experience. Additionally, the definition of SAEs varied across included trials, incorporating both procedure-related and systemic medical complications, which may introduce heterogeneity in pooled safety outcomes. Another limitation lies in the heterogeneity of DMVO definitions across studies, particularly with regard to the anatomy of the M2 segment of the MCA. The classification of M2 branches varies, which can lead to potential disparities in patient selection. Future studies should strive for anatomical clarity by explicitly defining DMVO as occlusion beyond the horizontal (M1) segment of the MCA, thereby improving comparability across trials and reducing definitional issues. Furthermore, the classification of codominant branches frequently focuses on anatomical size or flow capacity; however, this anatomical emphasis may fail to capture the functional importance of the perfused cerebral territory. Future research should incorporate not only anatomical dimensions but also the eloquence of the downstream brain regions, as this may have greater functional relevance when evaluating the potential benefit of EVT in DMVO. Notably, patients with aphasia or an NIHSS ≥ 8 in the anterior circulation may represent a subgroup at higher risk of disabling stroke, potentially benefiting more from thrombectomy [2, 29]. The low median NIHSS scores in current RCTs may have favored medical treatment, underscoring the need for more effective stratification in future trials. Additionally, the sICH rate reported in the DISCOUNT trial was higher than those other trials, which may reflect differences in patient selection, imaging criteria, or event classification. Lastly, although the data for the DISCOUNT [14] trial was publicly presented at the International Stroke Conference 2025, it has not yet been published in a peer reviewed journal so we performed a sensitivity analysis excluding it. This reanalysis supports the previous results and further enhances the generalizability and reliability of our conclusions.

## Conclusion

The present meta-analysis, which pools the available RCTs evaluating EVT plus BMT versus BMT alone in minor stroke, shows that EVT of DMVOs does not significantly improve prognosis and is actually associated with a greater risk of SAEs. These results align with the conclusions from the included RCTs and challenge previous evidence from observational studies that suggest a benefit from EVT in this patient population. Our results do not support EVT for DMVO and call for a reassessment of treatment guidelines to optimize patient selection criteria as well as to improve the quality of care and patient outcomes.

## Supplementary Information

Supplementary Material 1

## Data Availability

No datasets were generated or analysed during the current study.
