# Supplementary material for: Endovascular thrombectomy for distal medium vessel occlusion stroke: A meta-analysis of randomized controlled trials
Source: Neurosurg Rev. 2025 Oct 10;48(1):691. doi: 10.1007/s10143-025-03835-0 (PMC12513949; doi:10.1007/s10143-025-03835-0)

**Supplementary Online Content**

**SFigure 1.** Forest plot showed the meta-analysis of 90-day functional excellence (mRS 0-1)


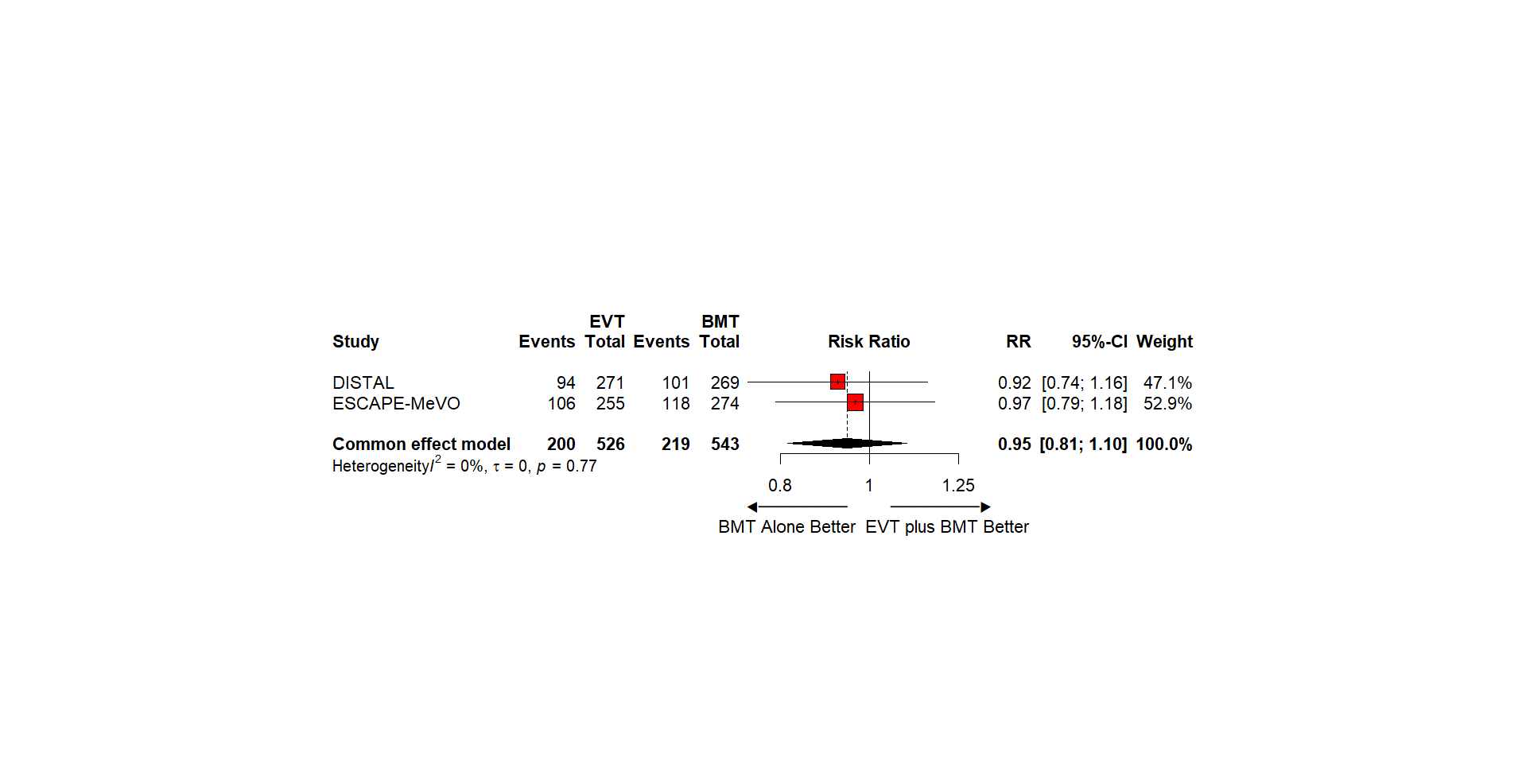


**SFigure 2.** Forest plot showed the meta-analysis of 90-day functional independence (mRS 0-2)


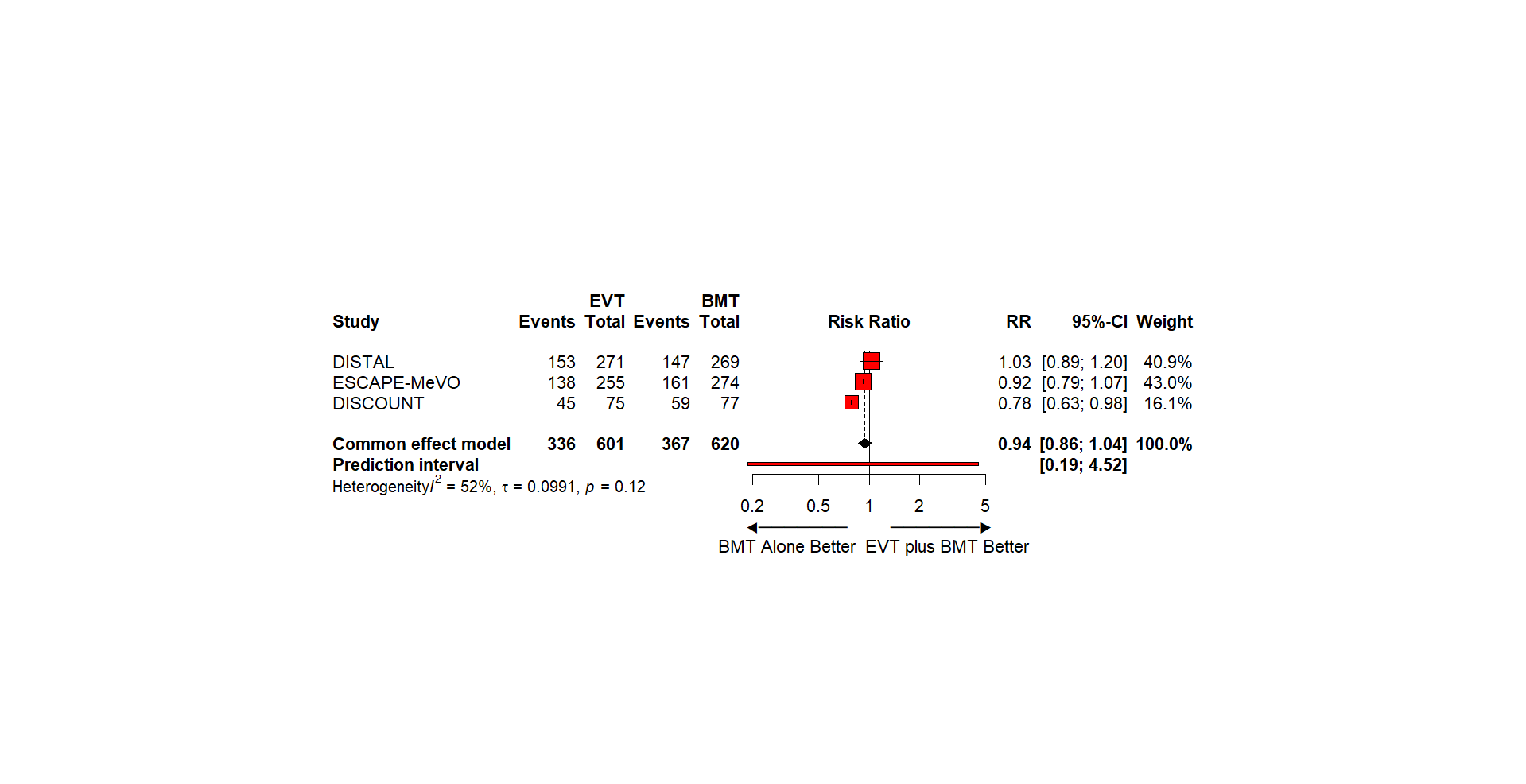


**SFigure 3.** Forest plot showed the meta-analysis of 90-day functional independence (mRS 0-3)


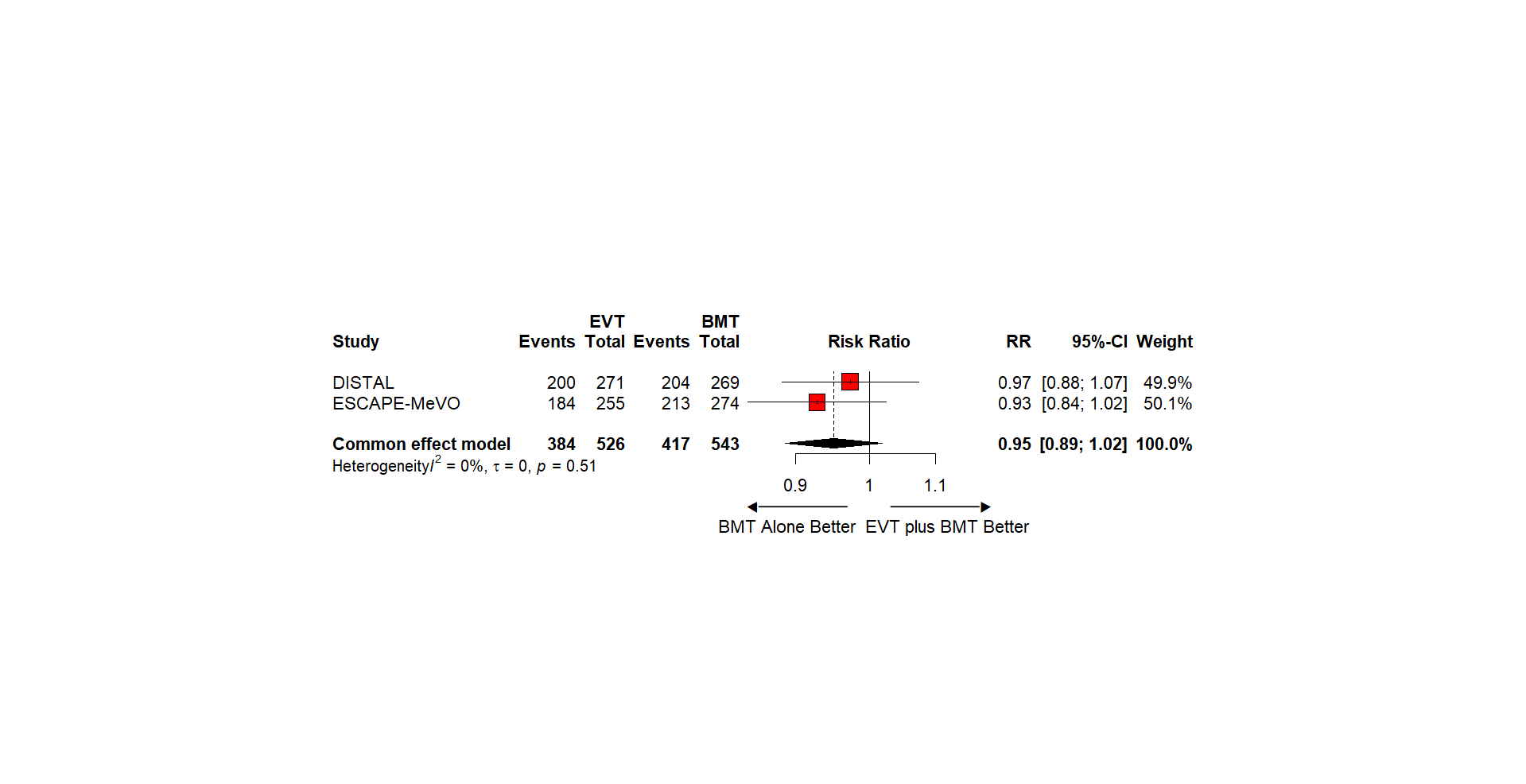


**SFigure 4.** Forest plot showed the meta-analysis of 90-day death or dependency (mRS 4-6)


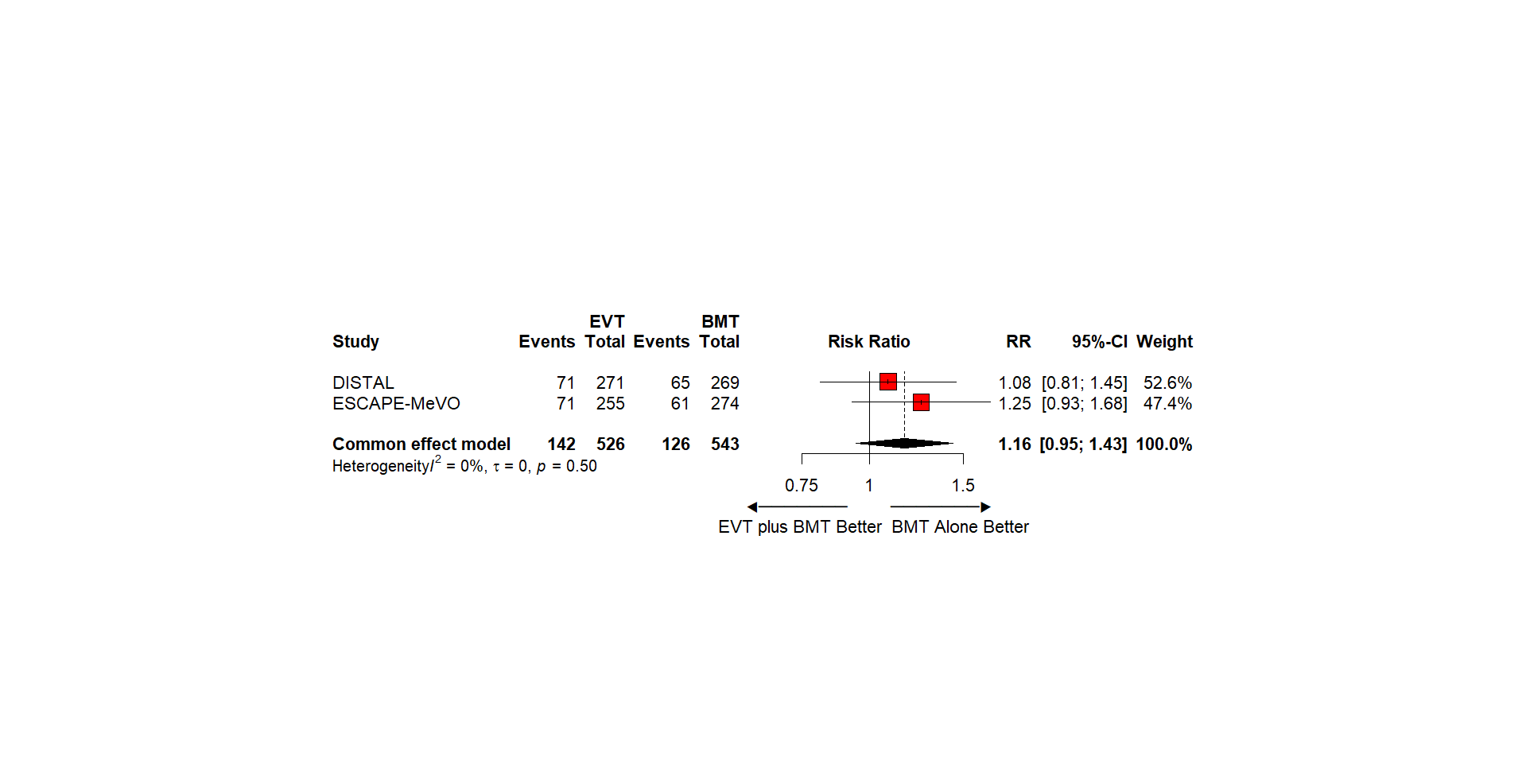


**SFigure 5.** Forest plot showed the meta-analysis of 90-day overall survival (mRS 0-5)


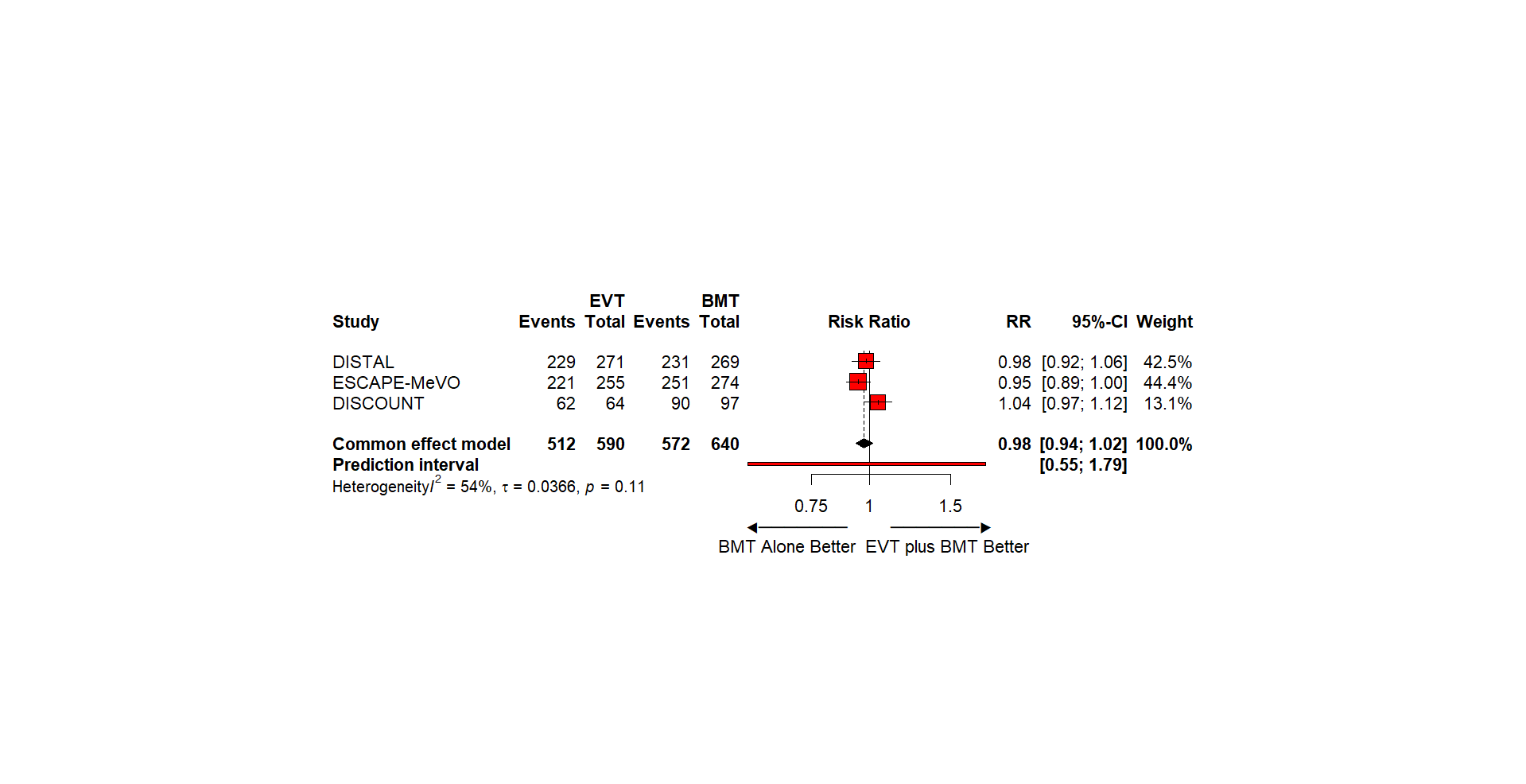


**SFigure 6.** Forest plot showed the meta-analysis of 90-day mortality (mRS 6)


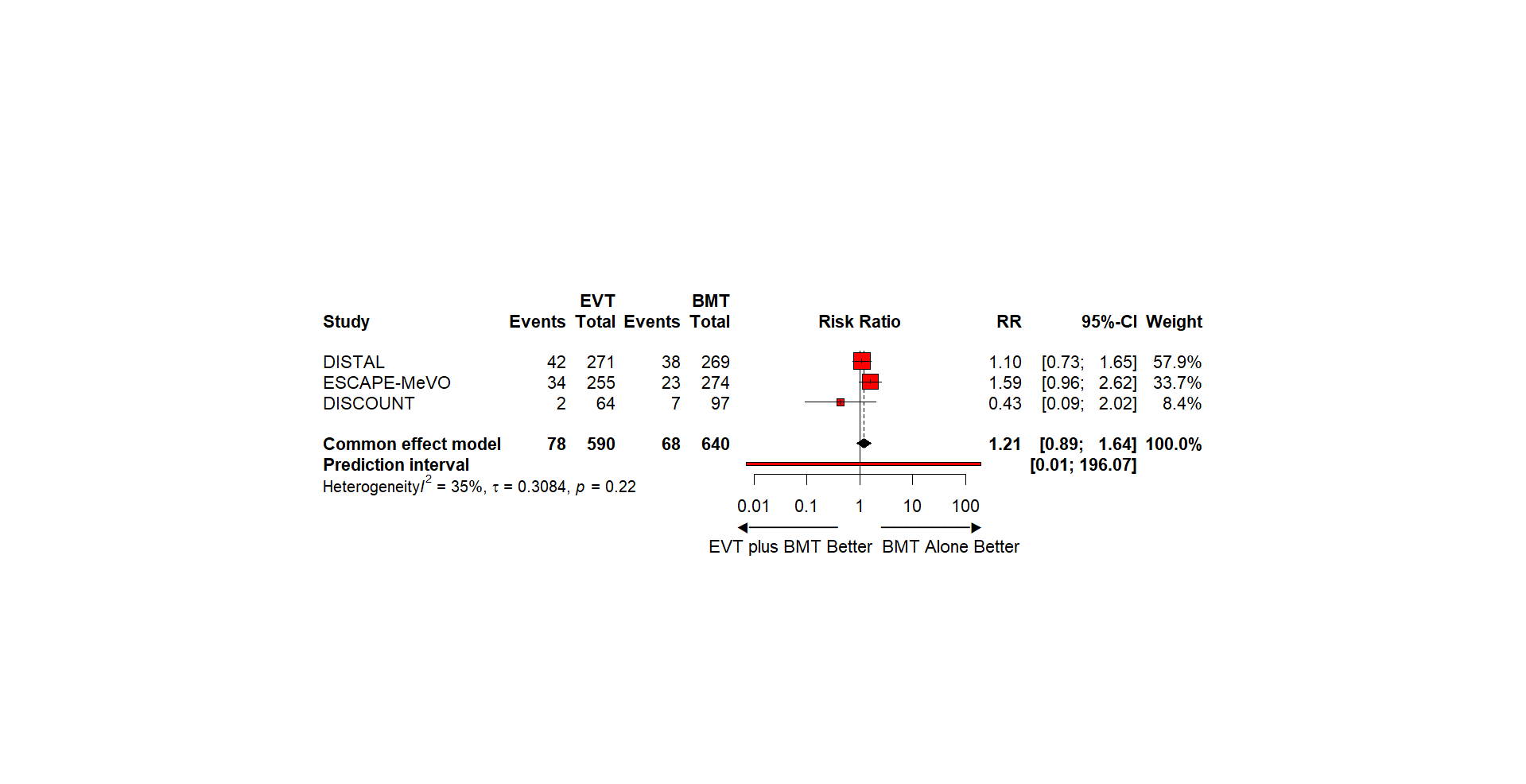


**SFigure 7.** Forest plot showed the meta-analysis of 90-day serious adverse events

**
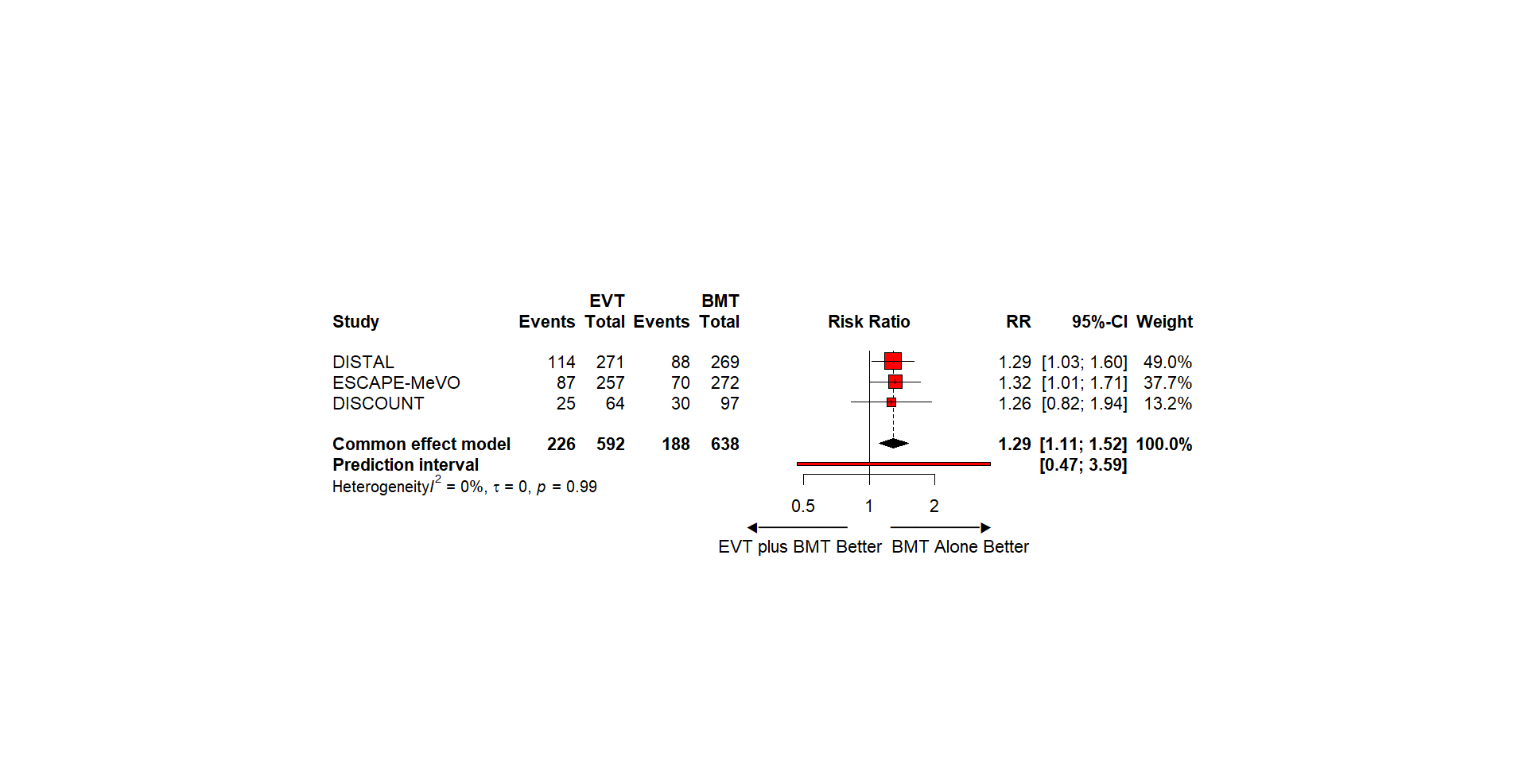
**

**SFigure 8.** Forest plot showed the meta-analysis of symptomatic intracranial hemorrhage


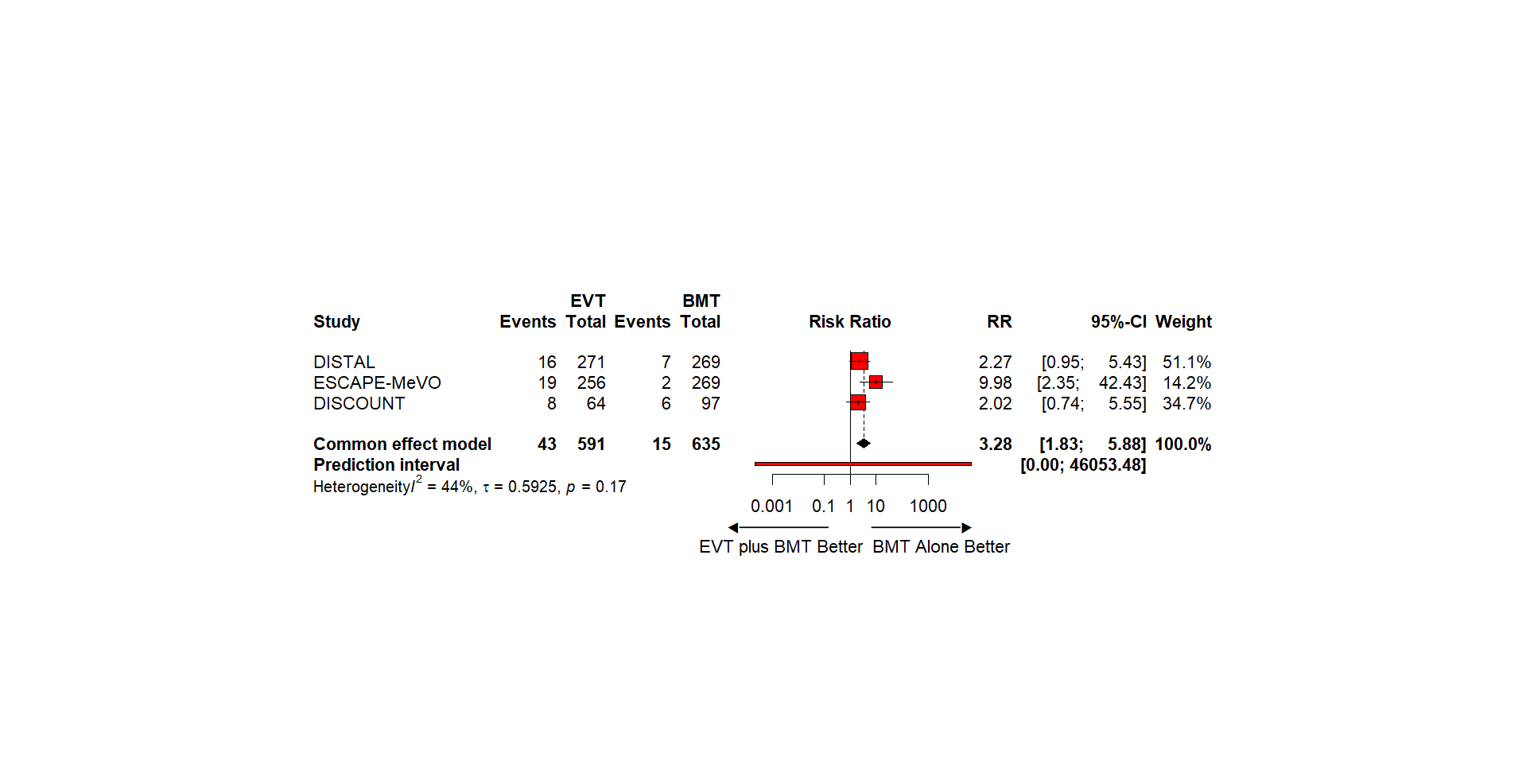


**SFigure 9**. Risk of Bias assessment traffic light plot


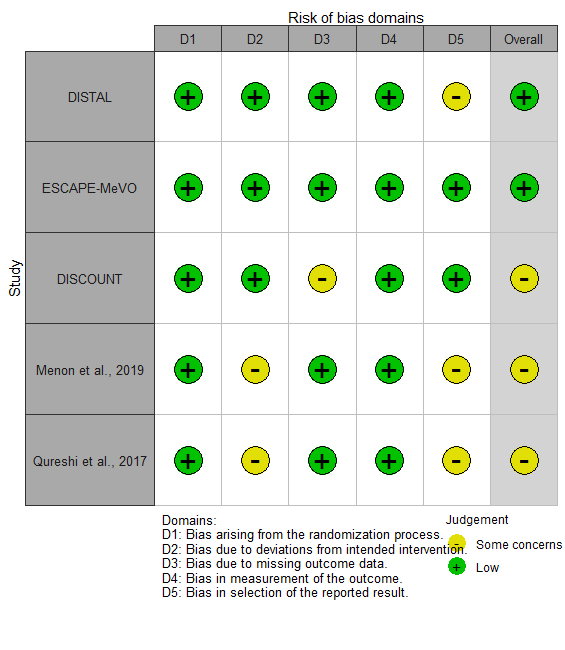


**SFigure 10**. Risk of Bias assessment bar plot


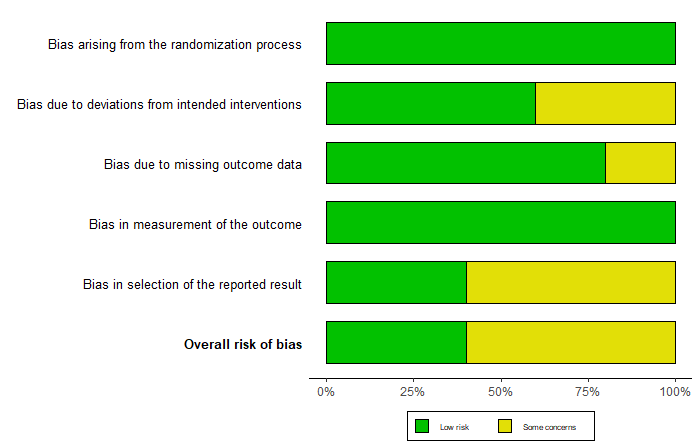

Supplement: Supplementary file 1 — Supplementary Material 1 [file 10143_2025_3835_MOESM1_ESM.docx]
